# Case report: Neonatal autoimmune lymphoproliferative syndrome with a novel pathogenic homozygous *FAS* variant effectively treated with sirolimus

**DOI:** 10.3389/fped.2023.1150179

**Published:** 2023-04-20

**Authors:** Fawzia M. Elgharbawy, Mohammed Yousuf Karim, Dina Sameh Soliman, Amel Siddik Hassan, Anoop Sudarsanan, Ashraf Gad

**Affiliations:** ^1^ Neonatal Intensive Care Unit, Department of Pediatrics, AL Wakra Hospital, Hamad Medical Corporation, Doha, Qatar; ^2^ Weill Cornell Medicine- Qatar (WCM-Q), Cornell University, Doha, Qatar; ^3^ Immunopathology Section, Sidra Medicine, Doha, Qatar; ^4^ College of Medicine, Qatar University, Doha, Qatar; ^5^ Department of Laboratory Medicine and Pathology, Hamad Medical Corporation, Doha, Qatar; ^6^ Allergy and Immunology section, Department of Pediatrics, Sidra Medicine, Doha, Qatar; ^7^ Neonatal Intensive Care Unit, Women's Wellness and Research Center, Hamad Medical Corporation, Doha, Qatar

**Keywords:** ALPS (autoimmune lymphoproliferative syndrome), DNT-cells, sirolimus, *FAS*, novel variant, newborn, autosomal recessive

## Abstract

**Background:**

Autoimmune lymphoproliferative syndrome (ALPS) is a rare disease characterized by defective *FAS* signaling, which results in chronic, nonmalignant lymphoproliferation and autoimmunity accompanied by increased numbers of “double-negative” T-cells (DNTs) (T-cell receptor αβ+ CD4−CD8−) and an increased risk of developing malignancies later in life.

**Case presentation:**

We herein report a case of a newborn boy with a novel germline homozygous variant identified in the *FAS* gene, exon 9, c.775del, which was considered pathogenic. The consequence of this sequence change was the creation of a premature translational stop signal p.(lle259*), associated with a severe clinical phenotype of ALPS-*FAS*. The elder brother of the proband was also affected by ALPS and has been found to have the same *FAS* homozygous variant associated with a severe clinical phenotype of ALPS-*FAS*, whereas the unaffected parents are heterozygous carriers of this variant. This new variant has not previously been described in population databases (gnomAD and ExAC) or in patients with *FAS*-related conditions. Treatment with sirolimus effectively improved the patient clinical manifestations with obvious reduction in the percentage of DNTs.

**Conclusion:**

We described a new ALPS-*FAS* clinical phenotype-associated germline *FAS* homozygous pathogenic variant, exon 9, c.775del, that produces a premature translational stop signal p.(lle259*). Sirolimus significantly reduced DNTs and substantially relieved the patient's clinical symptoms.

## Introduction

Autoimmune lymphoproliferative syndrome (ALPS), a rare hereditary immune regulatory condition that disrupts lymphocyte homeostasis, was genetically identified in 1995, when the first disease-causing *FAS* gene variants were found (1). Clinically, ALPS has been known since the 1960s (2) and is characterized by lymphoproliferation (lymphadenopathy and/or organomegaly), autoimmune symptoms (mostly cytopenia), and a high risk of developing lymphoma. Immunological tests typically reveal increased (T-cell receptor αβ+ CD4−CD8−) “double-negative” T-cells (DNTs), and other ALPS biomarkers, such as high levels of vitamin B12, interleukin-10 (IL-10), and soluble *FAS* ligand (*sFASL*), as well as a problem with *FAS*-mediated apoptosis (3). Noncancerous lymphoproliferation and cytopenia in pediatric patients with ALPS are usually treated with immunosuppressants such as steroids and sirolimus, while they are closely monitored for malignancy development (4).

## Case presentation

**Figure 1:**
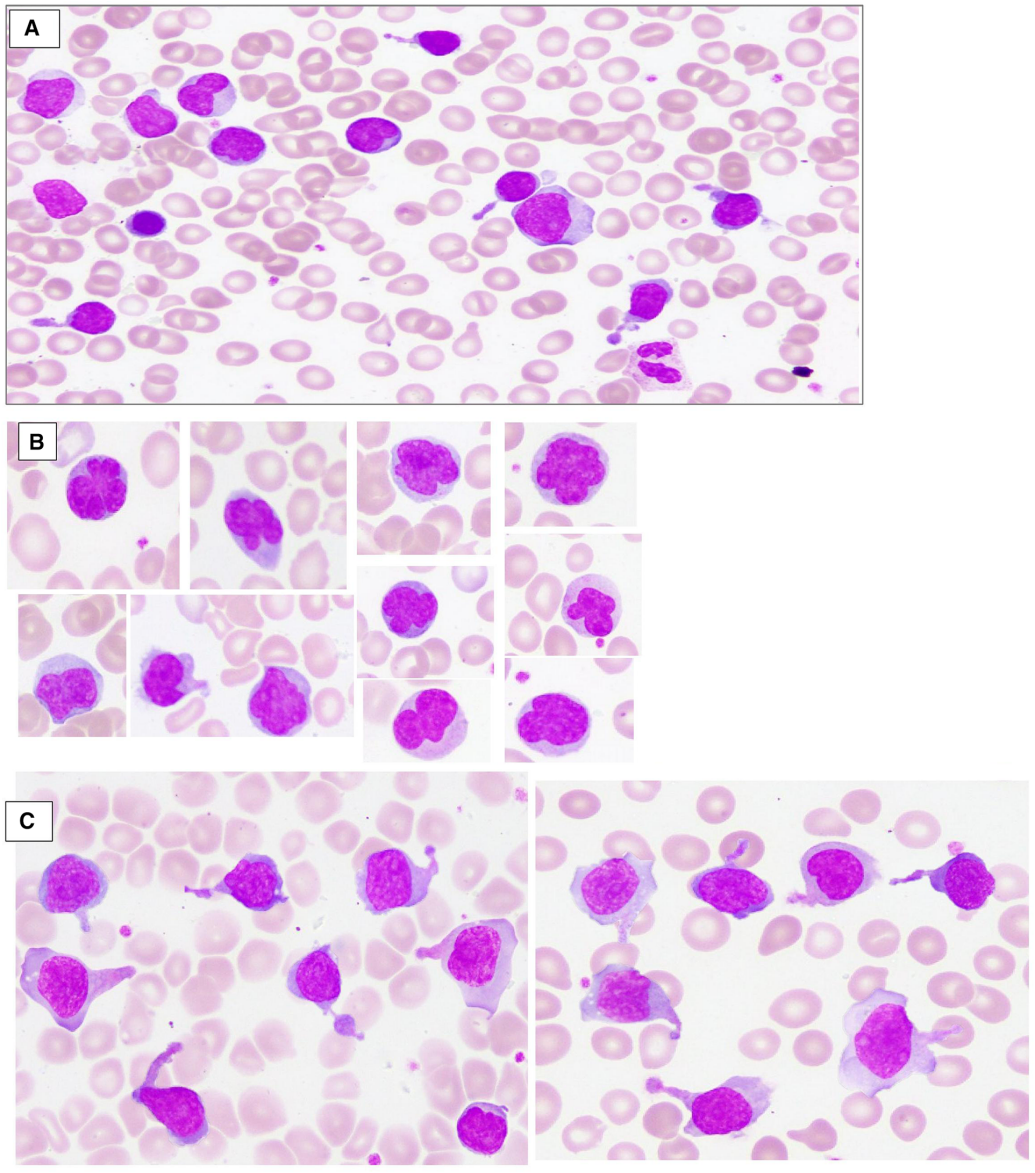
Peripheral blood smear, wright stain depicts moderate normocytic normochromic anemia with leukocytosis, and marked lymphocytosis composed of pleomorphic mature-looking lymphoid cells (**A**); many atypical forms showing marked nuclear irregularities with multilobated forms (flower-like cells) (**B**); and/or cytoplasmic projections/pseudopods (**C**).

A 3.9 kg-baby-boy was born *via* cesarean section at 37 weeks’ gestation to consanguineous parents. The mother was a 33-year-old fourth gravida with a history of one spontaneous first trimester miscarriage. Her first baby died at 10 days of age due to sepsis. The second child was diagnosed with ALPS at 1 year of age with the same pathogenic homozygous *FAS* variant. He presented with persistent lymphadenopathy, anemia, and hepatosplenomegaly at the age of 2 months. Both parents were heterozygous carriers of the *FAS* variant. There was a family history of ALPS in the maternal uncle, though we do not know the pathogenic variants. The infant was born vigorous, but developed respiratory distress shortly after birth, requiring noninvasive (NIV) respiratory support and neonatal intensive care unit (NICU) admission. The abdomen was markedly distended with hepatosplenomegaly on palpation; the liver edge was felt at 7 cm, and the spleen was 8 cm below the right and the left costal margins, respectively, but there was no lymphadenopathy. The complete blood count (CBC) showed leukocytosis with predominant lymphocytosis, anemia, and thrombocytopenia (serial CBCs are summarized in Table 1); the white blood cell count (WBC) at birth was 29 × 10^3^/µl, lymphocytes 20.6 × 10^3^/µl, eosinophils 0.6 × 10^3^/µl, hemoglobin (Hb) 12.2 g/dl, and platelets 73 × (10^3^/µl). Peripheral smear (Figure 1) showed moderate normocytic normochromic anemia with leukocytosis, significant eosinophilia, and marked lymphocytosis composed of pleomorphic mature-looking lymphoid cells (Figure 1A), with prominent atypical forms showing cytoplasmic projections/pseudopods (Figure 1B) and/or marked nuclear irregularities with multilobated forms (flower-like cells) (Figure 1C).

**Table 1:** A summary of complete blood count results at various time intervals following birth.

	At birth	2 weeks (PRED 0.5 mg/kg daily)	1 month (PRED 2 mg/kg daily)	3 months (PRED + sirolimus 0.5 mg daily)	5 months (sirolimus 0.5 mg and PRED tapering)	10 months (sirolimus 0.5 mg daily, PRED off)
WBC (10^3^/µl)	29 (9.0–30)	33.3 (5.0–20)	42 (5.0–20)	7 (5.0–19.5)	5.7 (6.0–18)	16.8 (6.0–16)
Lymphocytes (10^3^/µl)	20.6 (2.0–11)	27 (2.0–17)	34 (2.0–17)	3.6 (2.0–16.5)	3.2 (2.8–14.4)	12.2 (2.7–12)
Eosinophils (10^3^/µl)	0.6 (0.2–0.7)	2.3 (0.2–0.6)	5.2 (0.2–0.6)	0.1 (0.2–0.6)	0.1 (0.0–1.1)	0.6 (0.0–1.0)
RBC (millions/mm^3^)	3.8 (4.0–6.6)	2.7 (3.6–6.2)	2.6 (3.6–6.2)	6.7 (2.7–4.9)	7.9 (3.5–5.2)	8.2 (3.5–5.6)
Hemoglobin (g/dl)	12.3 (14.5–22.5)	8.1 (12.5–20.5)	6.5 (10–18)	12.3 (9.0–14)	11.6 (9.5–13.5)	11.3 (10.5–13.5)
Platelets (10^3^/µl)	73 (140–450)	102 (140–450)	82 (140–450)	129 (140–450)	201 (140–450)	177 (140–450)

WBC, white blood count; RBC, red blood cells; PRED, prednisolone. Age normal ranges are included in parenthesis.

Liver and renal function test results were normal. Abdominal ultrasonography (US) at 2 days of age confirmed hepatomegaly (7 cm with normal echogenicity) and splenomegaly (7.9 cm with increased echogenicity). The baby underwent normal brain US. Echocardiography for murmur evaluation revealed a tiny atrial septal defect and a small patent ductus arteriosus. He remained in the NICU for 4 weeks, requiring NIV support for 22 days. He received antibiotics for 2 weeks due to meningitis diagnosed on the basis of abnormal cerebrospinal fluid (CSF) parameters and high inflammatory markers, the mother tested positive for *Streptococcus agalactiae* but did not receive antibiotics prior to delivery. Although blood culture and CSF culture showed negative results, the antibiotic course was completed in view of immunocompromise related to suspected ALPS and the family history of neonatal death.

Based on the positive ALPS family history, clinical picture, and laboratory findings, along with Clinical Hematology and Immunology consults, he was started on prednisolone at 2 weeks old for presumed ALPS. CBC at that time revealed WBC 33.3 × 10^3^/µl, lymphocytes 27 × 10^3^/µl, eosinophils 2.3 × 10^3^/µl, Hb 8.1 g/dl, and platelets 102 × 10^3^/µl. Prednisolone was slowly increased to 2 mg at 1 month of age (Table 1).

**Figure 2:**
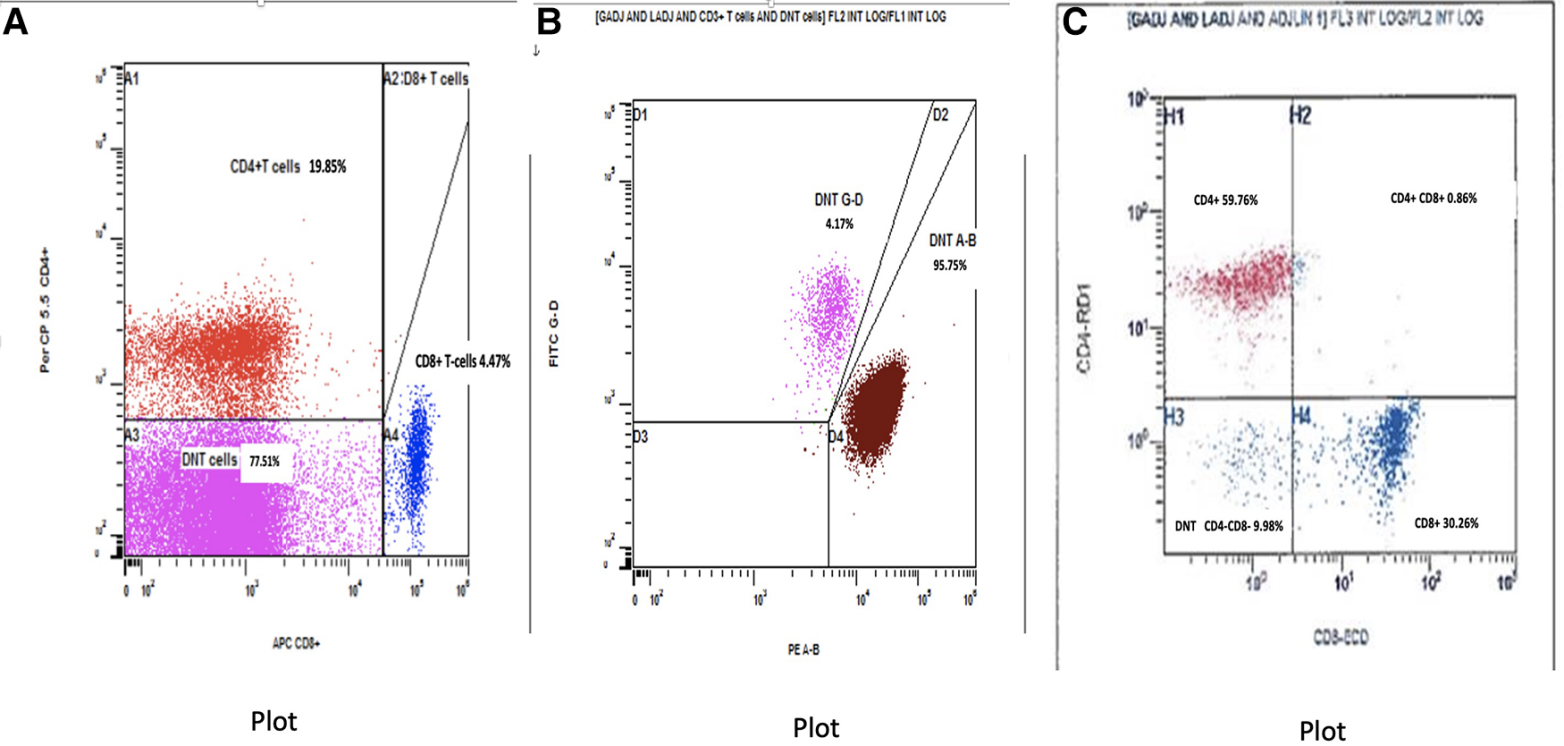
(**A**) Baseline flow cytometry plot gated on CD3+ T-cells to assess the expression of CD4 and CD8. Lower left quadrant indicates DNTs (including αβ and γδ T-cells). (**B**) Baseline flow cytometry plot gated on DNTs showing predominant αβ vs. γδ T-cell receptor expression. (**C**) Post-treatment flow cytometry plot gated on CD3+ T-cells to assess CD4 and CD8 expression. The huge population of DNT noted pretreatment in the lower left quadrant has almost completely disappeared. DNT, double-negative CD3+ T-cells.

The baby was formally diagnosed with ALPS after flow cytometry and genetic testing. Immunophenotyping for T-cell subsets (ALPS panel) performed at 6 weeks of age (Figure 2A,B) demonstrated a marked increase in CD4−CD8− αβ T-cells 23,429 cells/mcL, (NR 6–86 cells/mcL), representing 74.21% of total CD3+ T-cells (diagnostic criterion for ALPS >2.5%). Figure 2B, which was gated on the CD4−CD8− DNTs, confirmed that the DNTs were predominantly αβ-TCR-positive (95.75% of DNTs) rather than γδ-TCR-positive (4.17%). For further characterization of DNTs, the findings were corroborated by flow cytometry performed on peripheral blood at a different timepoint using pan T-cell markers (leukemia panel), revealing 57.5% (of total CD3+ T-cells) CD4−/CD8− αβ T-cells expressing pan T-cell markers (CD3, CD2, and CD5) and showing downregulation of CD7. The DNTs were positive for CD38 and negative for CD25 (data not shown).

**Figure 3:**
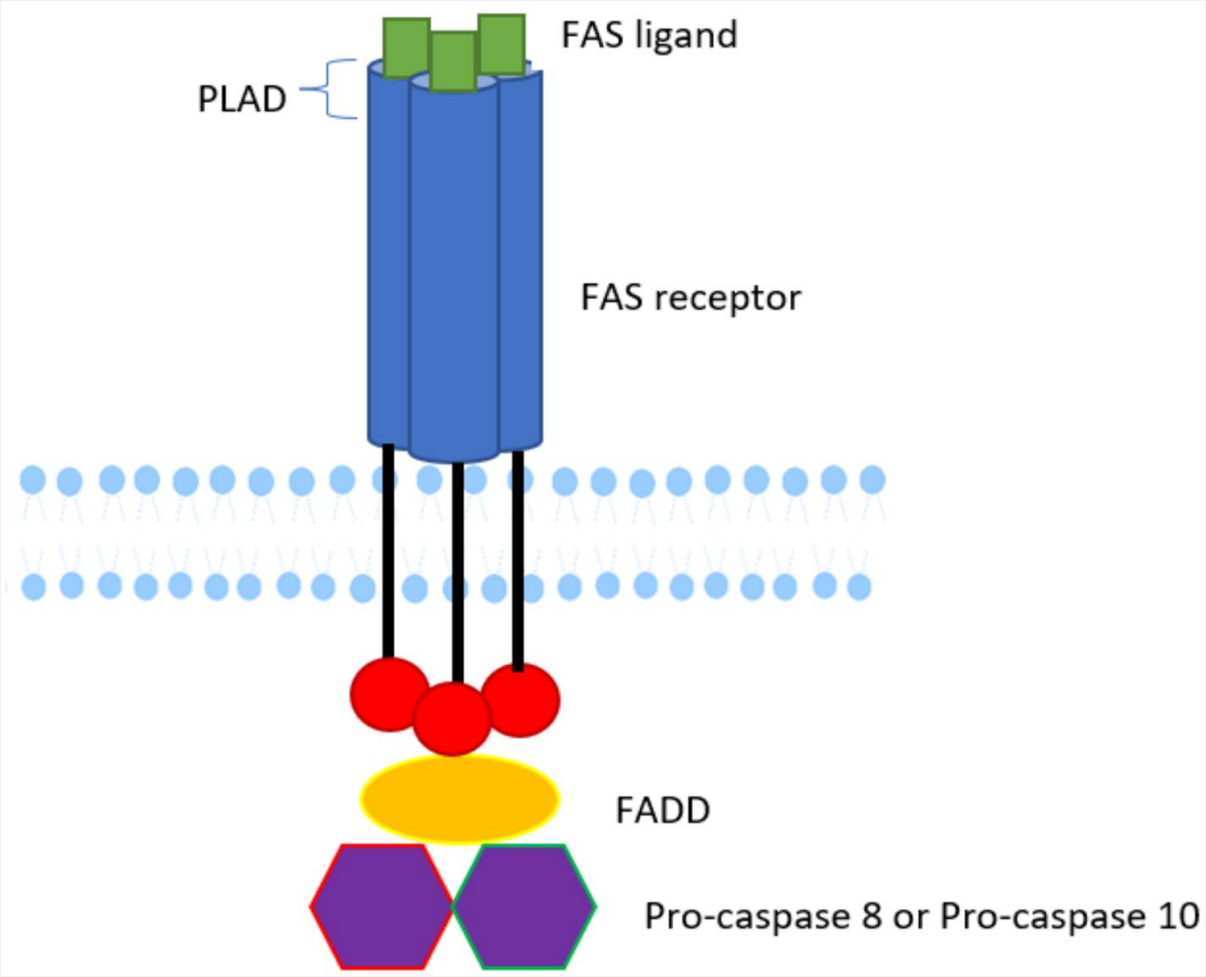
Schematic diagram of *FAS* protein. The structure of *FAS* protein is indicated. The pathogenic variant affects the death domain, consequently affecting the signal transduction pathway, which would normally involve recruitment of FADD and activation of pro-caspase 8 or 10. Diagram courtesy of Khadija Karim.

Whole exome sequencing (WES) performed at 6 weeks of age revealed a novel homozygous variant identified in the *FAS* gene, exon 9, c.775del p.(lle259*), based on the reference sequence transcript *FAS* NM_000043.6. The Phred combined annotation-dependent depletion (CADD) score was 31, strongly suggesting the variant is deleterious. This variant affects the death domain, consequently affecting the signal transduction pathway, which normally involves the recruitment of *FAS-*associated death domain (FADD) and the activation of pro-caspase 8 or 10 (Figure 3). This homozygous variant and the resulting putative change p.(Ile259*) in the death domain affecting our patient has not been previously reported (5). Previously, the elder brother affected by ALPS was found to have the same homozygous variants in *FAS*, whereas the unaffected parents were heterozygous carriers of the same variant. An uncle was also reported to be affected, but we do not have access to sequencing data.

The baby had a suboptimal response to prednisolone and developed systemic hypertension. Added to the experience of using sirolimus in his brother, who demonstrated remission after 4 months of treatment, he was started on sirolimus at 7 weeks of life at a dose of 0.5 mg once daily, with gradual tapering of prednisolone. The CBC revealed a significant improvement: after 1 month of sirolimus treatment, the WBC dropped to 7 × 10^3^/µl, lymphocytes 3.6 × 10^3^/µl, eosinophils 0.1 × 10^3^/µl, Hb 12.3 g/dl, and platelets 129 × 10^3^/µl. Immunoflow cytometry after 2 months of sirolimus revealed remarkable improvement. Total DNTs had reduced markedly from 77.51% of T-cells (NR 1.1%–4.0%) down to 9.98% (NR 3.1%–6.7%) (Figure 2C). At 5 months (i.e., receiving sirolimus for 3 months), an abdominal US showed normal liver and spleen size with normal echogenicity. At the same 5 month timepoint, the abnormal lymphocyte forms with cytoplasmic projections/pseudopods and the flower-like cells were no longer visible on the peripheral blood smear. By 7 months, there was further improvement in total WBC 5.7 × 10^3^/µl and lymphocyte count 3.2 × 10^3^/µl; full serial CBC parameters shown in Table 1. The baby was continued on a tapering dose of prednisolone and sirolimus 0.5 mg daily. At 10 months, prednisolone was withdrawn, with sirolimus 0.5 mg daily. The baby is being closely followed up, with regular CBC, peripheral smear, and lymphocyte subsets.

## Discussion

Here, we describe a case of ALPS in a term newborn who presented with marked hepatosplenomegaly during the immediate postnatal period. ALPS alters immunological homeostasis by affecting apoptosis, most commonly through dysfunction in the *FAS* apoptotic pathway. Over 70% of ALPS patients have a detectable variant in *FAS* pathway genes. Activated B and T-lymphocytes upregulate *FAS* expression, and T-lymphocytes upregulate *FAS* ligand expression. Defects in apoptosis result in lymphoproliferation, autoimmunity, and predisposition to malignancy. In most ALPS-*FAS* patients, heterozygous germline variants are inherited in an autosomal dominant (AD) manner (6). However, a minority of families demonstrate autosomal recessive inheritance. Indeed, in our patient, a novel homozygous variant identified in *FAS*, exon 9, c.775del, was considered pathogenic. The consequence of this sequence change is the creation of a premature translational stop signal p.(lle259*), which can potentially disrupt the last 77 amino acids of the *FAS* protein. Although this variant has not previously been described in population databases (gnomAD and ExAC) or in patients with *FAS*-related conditions, it is considered to be pathogenic on the basis of the clinical and biological considerations; the elder brother affected with ALPS had been found to have the same homozygous variant in *FAS*, while the unaffected parents are heterozygous carriers of this variant. Furthermore, the variant is located in exon 9, which encodes the so-called “death domain” of the *FAS* protein; the variant disrupts the *FAS* protein in a location affected by reported pathogenic variants. We acknowledge that without staining for surface *FAS* expression it remains unclear whether signaling is abrogated due to complete loss of protein expression, e.g., due to non-sense-mediated RNA decay, or due to defective signal transmission of the truncated *FAS* receptor.

While severe perinatal onset is rare in AD ALPS, homozygous pathogenic variants in the *FAS* gene are typically characterized by severe disease with perinatal onset, as also noted in our patient's clinical presentation (7). This is the first case of ALPS reported in a newborn with severe manifestations involving a new variant identified in *FAS*, exon 9, c.775del, but treated successfully with sirolimus. Other neonatal case reports of *FAS* pathogenic variants included two newborns presenting with hepatosplenomegaly (6, 8, 9), but the diagnosis of ALPS was not made until several months later in those infants, and neither were treated with sirolimus. In another report, the patient presented with intrauterine hepatosplenomegaly treated with sirolimus (10). In contrast to our infant, this child was started on a smaller dose of sirolimus (0.25 mg daily) and continued on concomitant prednisolone in the long term. To our knowledge, the distinct morphologic features of circulating lymphocytes demonstrated in our patient (Figure 1) have not been described in previous literature in ALPS patients (11). These morphologic findings significantly resolved after initial therapy with sirolimus, in parallel with reduction in DNT percentages by flow cytometry, which indicate that the abnormal DNTs are probably those with the abnormal morphology. However, the diagnostic and monitoring value of these peripheral smear findings warrants future confirmatory studies.

ALPS treatment targets symptoms and consequences. Although hematopoietic stem cell transplantation cures ALPS, it is usually reserved for severe and resistant cases (12). Spontaneous immunological cytopenias are treated with corticosteroids and intravenous immunoglobulins (13). Refractory cytopenia in ALPS patients sometimes require second-line treatments. Rituximab and/or mycophenolate mofetil (MMF) (14) were the first medications tested for thrombocytopenia, with the former being particularly helpful for thrombocytopenia but not usually advised due to a persistent increase in the risk of serious infections. MMF, on the other hand, has been shown to be an efficient steroid-sparing medication with no notable toxicities or infections (15).

Sirolimus is the only treatment shown to significantly reduce lymphoproliferation, consistent with the description of an overactive the mammalian target of rapamycin (mTOR) pathway in ALPS, which may be regarded as a targeted treatment for ALPS (16), commensurate with the high degree of remission attained and the large reduction of ALPS biomarkers after 6 months of medication. For these reasons, sirolimus may be regarded as a first-line therapy choice for such patients (17). As neonatal-onset ALPS is rare, there is insufficient data in the literature to compare the potential side effects from long-term sirolimus use (e.g., infections) with early hematopoietic stem cell transplantation (HSCT) in this patient group. Until further data are available, we would consider HSCT in early-onset ALPS to be reserved for patients with inadequate response to sirolimus and other medical therapies. There is a potential malignancy risk due to sirolimus, which has to be balanced with the intrinsic malignancy risk from ALPS.

## Patient perspective

Because the parents had two siblings with ALPS, they were concerned about the disease's prognosis and treatment options. They were dissatisfied with the side effects of prednisolone; however, they were pleased with the outcome of sirolimus treatment and the overall management provided by the multidisciplinary team.

## Conclusion

This is an inherited case of neonatal-onset ALPS with distinct morphological features on blood film that was successfully treated with sirolimus. ALPS should be considered in the differential diagnosis of newborns with massive hepatosplenomegaly. Early diagnosis, including flow cytometry and genetic testing, and treatment with sirolimus are crucial to achieving early remission and a better prognosis. Prompt genetic counseling should be considered for ALPS, especially if there is a family history of the disease.

## Data Availability

The datasets presented in this article are not readily available because of ethical and privacy restrictions. Requests to access the datasets should be directed to the corresponding author.
